# Genetic identification of mutations and MLST types associated with decreased susceptibility to ceftriaxone in *Neisseria gonorrhoeae*

**DOI:** 10.3389/fmicb.2025.1728860

**Published:** 2026-01-21

**Authors:** Xin Zhang, Hairui Wang, Yixin Gu, Xiaoli Chen, Guilan Zhou, Chang Liu, Liyin Ji, Rui Xie, Jianzhong Zhang, Zhujun Shao, Maojun Zhang

**Affiliations:** 1 National Key Laboratory of Intelligent Tracking and Forecasting for Infectious Diseases, National Institute for Communicable Disease Control and Prevention, Chinese Center for Disease Control and Prevention, Beijing, China; 2 Department of Clinical laboratory of Shengli Oilfield Central Hospital, Dongying Key Laboratory of Cell Biology, Dongying, China; 3 Nanjing Center for Disease Control and Prevention, Nanjing, Jiangsu Province, China

**Keywords:** decreased susceptibility to ceftriaxone, multilocus sequence typing (MLST), mutation frequencies, mutations, *Neisseria gonorrhoeae*, *penA* (penicillin-binding protein 2)

## Abstract

Antimicrobial resistance (AMR) in *Neisseria gonorrhoeae* severely limits treatment options, with increasing resistance even to first-line and last-line ceftriaxone (CRO), posing a major global public health threat. In this study, we systematically identified 53 significantly different mutations between ceftriaxone-resistant and susceptible strains in multiple proteins through bioinformatics analysis. Among these, 33 mutations were identified for the first time, notably including the PorB Q143K via structural analysis. Minimum spanning tree (MST) analysis based on these mutations marked improved sensitivity and specificity for identifying ceftriaxone-resistant strains compared to traditional sequence typing of PenA, PonA, PorB, and MtrR (68.4% vs. 53.2%; 77.3% vs. 57.5%, respectively). Furthermore, analysis of PenA sequences from global 8,325 strains (470 MLST types) revealed that mutation frequencies at key PenA sites are highly associated with MLST types, with 34 high-frequency MLST types (STs) identified. The proportions of these 34 STs were 88.38% in 611 decreased susceptibility to ceftriaxone (CRO-DS) strains and 33.09% in 8,325 background strains, respectively, revealing an extremely significant association between 34 high-frequency STs and CRO-DS (*P* < 0.0001). In conclusion, this work provides further insights into the molecular mechanisms of CRO resistance while offering significant value for monitoring and predicting emerging CRO-DS-associated MLST types.

## Introduction

*Neisseria gonorrhoeae* is the causative agent of gonorrhea, a globally prevalent sexually transmitted infection (STI), with approximately 82.4 million new infections in 2020 according to the World Health Organization (WHO) (WHO, 2024). The development of AMR in *N. gonorrhoeae* has narrowed treatment options, making ceftriaxone monotherapy or its combination with azithromycin the first-line and last-line treatments for uncomplicated gonorrhea (Workowski and Berman, 2010). However, in 2020, to address rising AMR to azithromycin, combination therapy was replaced by ceftriaxone monotherapy (500 mg, or 1 g for people ≥ 150 kg) in the United States (Cyr et al., 2020). Alarmingly, CRO-DS isolates have surged globally (Lin et al., 2022; Fifer et al., 2024; Xiu et al., 2024), and treatment failures due to high-level resistance have occurred (Unemo et al., 2012). A study of 4,113 *N. gonorrhoeae* isolates from Guangdong, China, found the prevalence of CRO-DS increased from 2.05% in 2016 to 16.18% in 2019 (Lin et al., 2022), highlighting the critical importance and urgency of CRO resistance surveillance and resistance mechanism research.

Ceftriaxone resistance in *N. gonorrhoeae* is multifactorial, with the genes *penA* (penicillin-binding protein 2), *ponA* (penicillin-binding protein 1A), *porB* (trimericporin PorB.IB), and *mtrR* (multidrug efflux system transcriptional repressor MtrR) being the most frequently analyzed and reported contributors (Lindberg et al., 2007; Lin et al., 2021; Liao et al., 2024). A few studies also report potential links between CRO-DS and genetic locus like the *mtrCDE* operator (efflux transporter MtrCDE operator) (Beggs et al., 2021), *bla*TEM (beta-lactamase TEM) ADDIN EN.CITE (Agbodzi et al., 2023; Qin et al., 2024), *rpoB* (DNA-directed RNA polymerase subunit beta), *rpoD* (RNA polymerase sigma factor RpoD) (Palace et al., 2020), and *rpoH* (RNA polymerase sigma factor RpoH) (Beggs et al., 2021). PenA is the paramount determinant of CRO resistance in *N. gonorrhoeae*, with two distinct types: non-mosaic and mosaic alleles acquired through horizontal gene transfer (Unitt et al., 2024). For non-mosaic *penA*, structural analysis shows multiple mutations at A501 increase resistance to CRO. Transformation with the A501T mutation increased CRO MIC twofold, statistically confirmed (Zhu et al., 2023). Similarly, transforming mosaic *penA* with A501V significantly increased CRO MIC (Tomberg et al., 2010). Additionally, G542S and P551L/S are also associated to CRO-DS (Whiley et al., 2010). For mosaic *penA*, A311V and T483S are reported to associate with CRO-DS (Liao et al., 2024). Furthermore, A311V serves as a key marker for detecting FC428-like mosaic strains (Wang et al., 2024).

Molecular typing is critical for studying genetic evolution and antimicrobial resistance surveillance in *N. gonorrhoeae*. MLST (multilocus sequence typing), NG-STAR (*Neisseria gonorrhoeae* Sequence Typing for Antimicrobial Resistance), and NG-MAST (*Neisseria gonorrhoeae* multi-antigen sequence typing) are the three primary typing schemes (Smolarczyk et al., 2021). MLST compares genetic variations in seven conserved housekeeping genes (abcZ, adk, fumC, aroE, pdhC, gdh, pgm) and exhibits high discriminatory power for global isolates (Bennett et al., 2007). The NG-STAR website (https://ngstar.canada.ca) offers a standardized method of classifying seven well-characterized genes (*penA, mtrR, porB, ponA, gyrA, parC* and 23S *rRNA*) associated antimicrobial resistance to three classes of antibiotics (cephalosporins, macrolides and fluoroquinolones) (Demczuk et al., 2017). NG-MAST is a highly discriminatory molecular typing method targeting a 490-bp *porB* fragment and a 390-bp *tbpB* (transferrin-binding protein subunit B) fragment (Bilek et al., 2007).

An increasing number of studies, especially in Asian regions, have associated CRO-DS strains with specific MLST types (STs). In Shenzhen, China (2014–2018), ST7365 (21.43%, 12/56) and ST7360 (18.18%, 10/55) significantly correlated with CRO-DS (Li et al., 2021). Across 20 cities in Guangdong, China (2021), CRO-DS was mainly associated with ST7363 (16%, 8/50), ST1903 (14%, 7/50), ST1901 (12%, 6/50), and ST7365 (10%, 5/50) (Liao et al., 2023). Among 463 isolates from Guangdong, Jiangxi, and Shanghai, China (2021), an 8.9% CRO-DS rate was detected, highly linked to ST7365 and ST1903 (Tang et al., 2023). In Hanoi, Vietnam (2023–2024), the CRO resistance rate was 27% (90/352 isolates), with ST1901 predominating (71.1%, 64/90) (Laumen et al., 2025). However, the reason why these specific STs are consistently linked to CRO-DS remains unclear. It appears not to be due to their high prevalence. For example, ST9363, one of the most globally prevalent STs, is rarely linked to CRO-DS, with reports mainly related to resistance to agents like azithromycin (Melendez et al., 2024). Therefore, this study systematically analyzed the genetic determinants of CRO resistance, MLST types, and CRO-DS in *N. gonorrhoeae* to investigate the molecular mechanisms of CRO resistance and identify STs associated with CRO-DS.

## Materials and methods

### Strain collection and screening

As of November 18, 2024, a total of 23,381 whole-genome sequences of *N. gonorrhoeae* isolates from 2005 to 2024 were downloaded from PubMLST database (https://pubmlst.org/) (Jolley et al., 2018). The European Committee on Antimicrobial Susceptibility Testing (EUCAST) defines an MIC of ≤ 0.125 mg/L as susceptible and an MIC of > 0.125 mg/L as resistant to ceftriaxone in *N. gonorrhoeae* (EUCAST, 2025). Among these, 94 isolates were CRO-R strains, 14,118 isolates were CRO-S strains, and 9,169 isolates had no available CRO resistance information.

Genomes used for the analysis of genetic determinants of CRO resistance were required to have ≤ 300 scaffolds and ≤ 1,000 N bases, resulting in the selection of 79 CRO-R strains. For CRO-S strains, 10 isolates were selected annually, preferably from different countries, to ensure geographic diversity. Due to the unavailability of CRO resistance data in 2023 and 2024, a total of 180 CRO-S strains were randomly selected. Including the susceptible reference strain FA1090, a total of 260 strains were selected (For detailed background information, see Supplementary Table 1). The genomes used for analyzing the correlation between PenA mutations and MLST types were selected from the 9,169 isolates not used in the previous analysis, all lacking cephalosporin resistance data. From these, we chose a total of 8,325 isolates with complete MLST information (for detailed background information, see Supplementary Table 2).

All the genomes of isolates were validated with the species identification module (taxonomy_wf) in Genome Database Taxonomy toolkit (GTDB-Tk) (Parks et al., 2022). All isolates were compared with a representative *N. gonorrhoeae* genome, and the average nucleotide identity (ANI) values were calculated for each isolate. A threshold of 0.95 ANI was applied to confirm that the genomes belonged to the *N. gonorrhoeae* species. The quality of the genomes was assessed using CheckM software v1.2.2 (Parks et al., 2015). To ensure the reliability of the genomes used for analyzing mutation sites, we applied stringent quality control criteria. Only the isolates with a completeness of ≥ 90% and contamination of ≤ 10% were considered suitable for further analysis.

### Phylogenetic and pan-genome analysis

Core Single Nucleotide Polymorphism (SNP) calling was conducted using the Snippy 4.6.0 (https://github.com/tseemann/snippy), utilizing FA1090 (GenBank accession no. AE004969.1) as a reference strain. The Gubbins 2.4.1 was employed as the recombination-removal tool, enabling the extraction of pure SNP data by eliminating recombination events. The genomes underwent gene prediction and functional annotation using the Prokka pipeline v1.14.6 (Seemann, 2014). Core-pan genome analysis was conducted using the Roary pipeline 3.13.0 (Page et al., 2015), which utilizes annotated assemblies in GFF3 format obtained from Prokka results. The MAFFT 7.471 was then employed to perform multiple sequence alignment of these sequences. Subsequently, the phylogenomic tree was constructed using FastTree 2.1.10 (Price et al., 2009) with the maximum-likelihood (ML) algorithm. The phylogenomic tree was visualized using iTOL v7.1.1 (Letunic and Bork, 2024).

### Analysis of genetic determinants of ceftriaxone resistance

Based on the reference sequence of each gene, a python script was used to extract the full-length amino acid sequences of the PenA, PonA, PorB, MtrR, *bla*TEM, RpoB, RpoD, RpoH, and a 27mer of *mtrCDE* operator, from 260 strains via BLAST alignment. Protein structure analysis was performed to identify key regions of each gene. AlphaFold 3 was used for 3D structure prediction with consistent seed values across all predictions to ensure comparability (Abramson et al., 2024). The crystal structure of the PenA-CRO complex was obtained from the PDB database (ID: 6XQV) (Fenton et al., 2021). Active pockets were predicted using POCASA 1.1 (Yu et al., 2010), and surface hydrophobicity was analyzed with the Python script color_h.py (Eisenberg et al., 1984). Structural alignment and visualization were carried out using PyMOL 2.5.2. WebLogo-3.7.9 was used to perform amino acid conservation analysis (Crooks et al., 2004). DeepPBS (Mitra et al., 2024) was used for geometric deep learning of protein-DNA binding specificity, with the parameter “Calculate heavy atom relative importance (RI) scores” enabled.

The 260 strains were divided into CRO-R (*n* = 79) and CRO-S (*n* = 181) groups. A python script was used to calculate the amino acid or nucleotide frequency at each site for each gene in both groups. For the *penA*, mosaicism was defined by alterations in amino acids 375–377, with mosaic and non-mosaic variants analyzed separately (Deng et al., 2019). Then, to identify mutation sites associated with CRO resistance or susceptibility, a two-tailed Fisher's exact test was performed for each differential site by R 4.4.1. A *P* value < 0.05 was considered statistically significant. Corrected *P* value (*q* value) was obtained using the Benjamini–Hochberg false discovery rate (FDR) approach. The Cleveland dot plot was generated using the R package tidyverse.

### Molecular typing

MLST, NG-STAR and NG-MAST (v2.0) were defined by using the PubMLST database. The cgMLST scheme of 260 strains was prepared using chewBBACA v2.8.5 (Silva et al., 2018). A training file generated by Prodigal v2.6.3 using the genome FA1090. The RemoveGenes module was used to delete the detected 39 paralogous loci from the 2,741 loci. The TestGenomeQuality module was used to verify the reliability of the dataset quality for 260 genomes. The ExtractCgMLST module was run to determine the set of loci in the core genome for the loci presence thresholds of 99%. Core genome composed of 1361/2702 genes. All minimum spanning trees used for molecular typing were constructed using the MSTreeV2 algorithm and visualized using GrapeTree v2.1 (Windows version) (Zhou et al., 2018). Statistical analysis of mutation sites was performed using the Chi-square or Fisher's exact test, with significance assessed by the false discovery rate (FDR) and defined by *q* values: *P q* < 0.05 as statistically significant, *q* < 0.01 as highly statistically significant, and *q* < 0.001 as extremely statistically significant.

### Analysis of the correlation between mutation frequencies at key PenA sites and MLST types

Eight thousand three hundred and twenty five genomes were annotated using Prokka, and sequences named “penicillin-binding protein 2” were exported from the faa files, resulting in 7678 non-mosaic *penA* alleles and 647 mosaic *penA* alleles. A cluster heatmap of PenA mutations sites and MLST types was generated using the R package pheatmap. A neighbor-joining tree was constructed using the FastME V2.0 algorithm in the GrapeTree software. The 647 mosaic *penA* alleles were assigned identification numbers through the pubMLST website. The nucleotide sequences of the 31 obtained types were then used to construct a maximum-likelihood tree using FastTree. A Sankey diagram of MLST, NG-STAR, and NG-MAST was created using the R package networkD3.

## Results

### Phylogenetic and pan-genome analysis of 260 strains employed for genetic determinants of ceftriaxone resistance analysis

Phylogenetic analysis of the 260 strains was conducted to trace evolutionary traits. Continent, country, gender, source, year, *penA* alleles, MLST, NG-STAR, NG-MAST, CRO-SIR (Susceptible, Intermediate, Resistant), CRO-MIC, CFM-SIR, CFM-MIC, and *porB* type are shown in Supplementary Figure 1. The 260 strains from 35 countries across six continents comprised 79 STs (ST1901, ST7363, ST9363, and ST1903 with ≥ 10 isolates each), 141 NG-STAR types, and 154 NG-MAST types.

**Figure 1:**
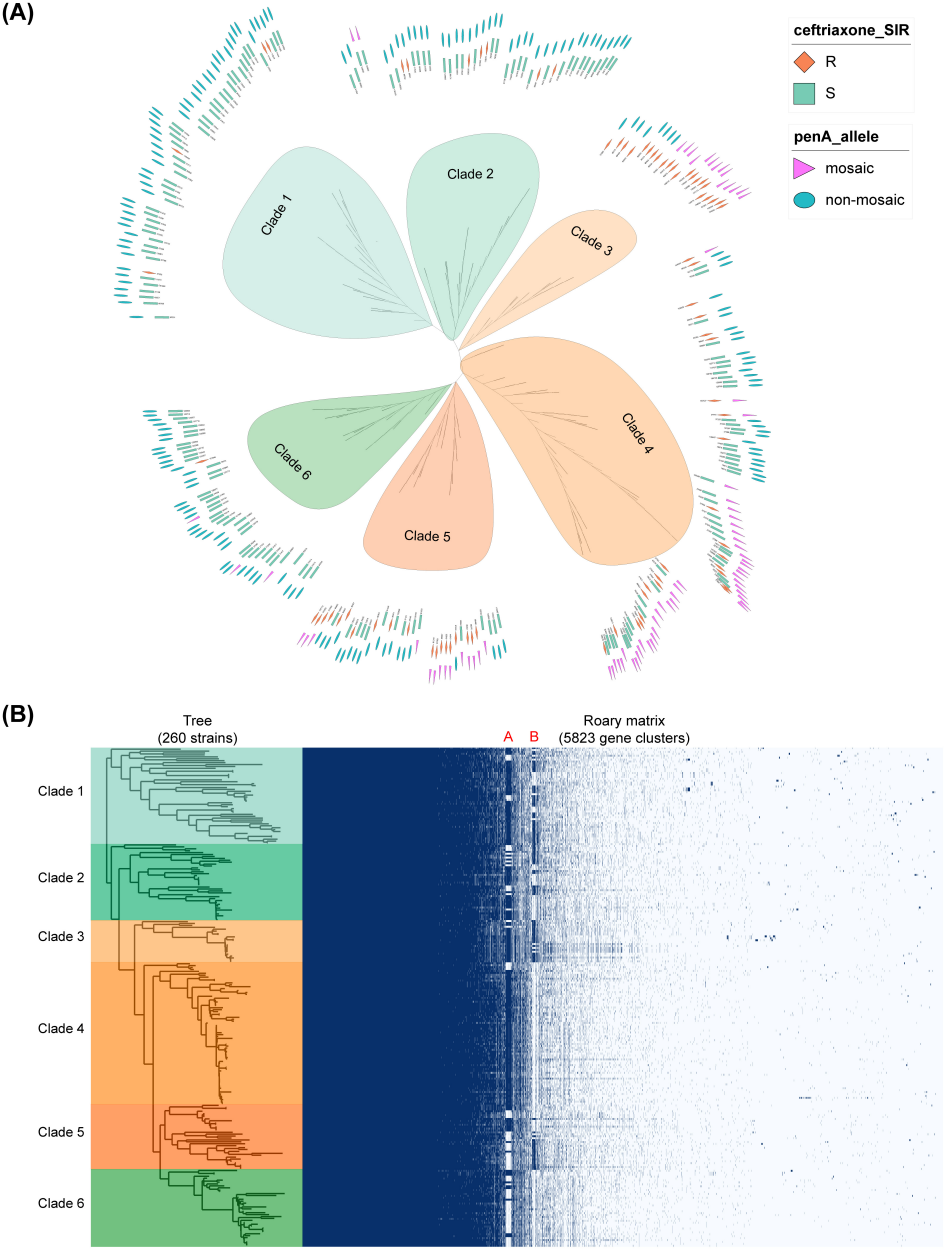
Phylogenetic and pan-genome analysis. **(A)** The phylogenetic tree based on core genes. The first layer annotation represents ceftriaxone SIR (Susceptible, Intermediate, Resistant), and the second layer annotation represents *penA* mosaic types. **(B)** Pan-genome matrix. Blue indicates presence and white indicates absence. Clade color coding applies to both **(A)** and **(B)**. Clades 3, 4, and 5 are represented in shades of orange, while Clades 1, 2, and 6 are shown in shades of green.

Core-pan genome analysis was used to explore the genetic diversity of 260 strains, defining core genes as those present in at least 99% of the strains with ≥ 95% identity. The pan-genome contained a total of 5,823 genes, including 1,481 core genes. According to the topology of the phylogenomic tree based on core genes, these strains can be divided into six clades, named Clade 1 to Clade 6. Interestingly, most ceftriaxone-resistant strains (67/79, 84.81%) and *penA* mosaic strains (74/79, 93.67%) clustered in Clades 3, 4, and 5 (Figure 1A). In combination with the phylogenomic tree constructed from core homologous clusters, these six clades exhibited significantly different gene family structures from each other, especially in the gene clusters labeled in red as A and B (Figure 1B). Clade 3 tends to possess both clusters, Clade 4 tends to have A but not B, whereas Clade 6 tends to lack both.

Further characterization identified a total of 73 genes (a = 47, b = 26) in the two clusters. The A cluster includes topA_2 (type I DNA topoisomerase), traC (type-IV secretion protein), parA (chromosome partitioning protein), dsbC (DsbC family protein), and yhaV (Ribonuclease toxin). The B cluster contains hin (DNA-invertase), traG (conjugal transfer protein), ssbB (single-stranded DNA-binding protein), trfA (plasmid replication initiator), trbM (Dutch type tetM conjugative plasmid), and traJ (conjugal transfer regulator). The remaining 62 genes are annotated as hypothetical proteins. Among the 11 identified genes, traC, parA, dsbC, traG, and ssbB are located on the horizontally transferred gonococcal genetic island (GGI, containing approximately 62 CDSs), and traC, parA, dsbC, and traG belong to the type IV secretion system genes encoded by GGI. In addition, the A cluster has a GC content of 45.1%, close to the reported GGI value of 44% (Hamilton et al., 2005), suggesting it contains more GGI-related genes. The B cluster has a GC content of 50.6%, close to the *N. gonorrhoeae* genome average of 51% (Dillard et al., 2011), indicating relatively fewer GGI-related genes.

### Analysis of genetic determinants of ceftriaxone resistance

Based on literature review, we identified 8 proteins and one operator potentially related to CRO-R in *N. gonorrhoeae*. We conducted structural analysis to pinpoint the regions or sites most impactful for CRO resistance. The structure of PenA is divided into two parts, with Q241-L564 being the region directly interacting with CRO, thus requiring special focus, especially around the active site S310 (Supplementary Figure 2A). Further interaction analysis identified 11 amino acids forming hydrogen bonds with CRO: E307, A310 (S310), S362, N364, Y422, T483, T498, T500, R502, G546, and V547 (Supplementary Figure 2E). Notably, there are 33 amino acids within 6Å of CRO: E307, P308, G309, A310 (S310), A311, K313, R345, T347, H348, K361, S362, N364, T417, F420, G421, Y422, T483, K497, T498, G499, T500, A501, R502, K503, Y509, H514, T517, Y543, Y544, G545, G546, V547, V548. Additionally, PenA includes non-mosaic and mosaic types, differing by 40-50 amino acids in the Q241-L564 region. Their structures, active pocket locations (Supplementary Figure 2B), surface charges, and hydrophobicity (Supplementary Figures 2C, D) also differ significantly. Therefore, mutation sites in mosaic and non-mosaic PenA must be analyzed separately.

The standard NG-STAR typing for PorB only covers 10 amino acids (positions 117–126, Supplementary Figure 2F, purple). However, structural analysis reveals a core variable region of 49 amino acids in the PorB channel (Supplementary Figure 2F, yellow + purple; sequences and conservation in Supplementary Figure 2G), with significant structural differences between PorB1b and PorB1a, the latter associated with disseminated gonococcal infection (Cartee et al., 2022). We therefore recommend expanding PorB1b analysis to include these 49 amino acids. Meanwhile, most studies focus on MtrR and the A deletion in its promoter's−35 region, but the mtrCDE operator region (including the−35 region), which directly binds the inhibitory protein, also warrants attention. DeepPBS was used to analyze the binding specificity between MtrR and the mtrCDE operator, identifying 10 directly interacting amino acids and multiple key specificity-determining nucleotide positions (Supplementary Figure 2H).

**Figure 2:**
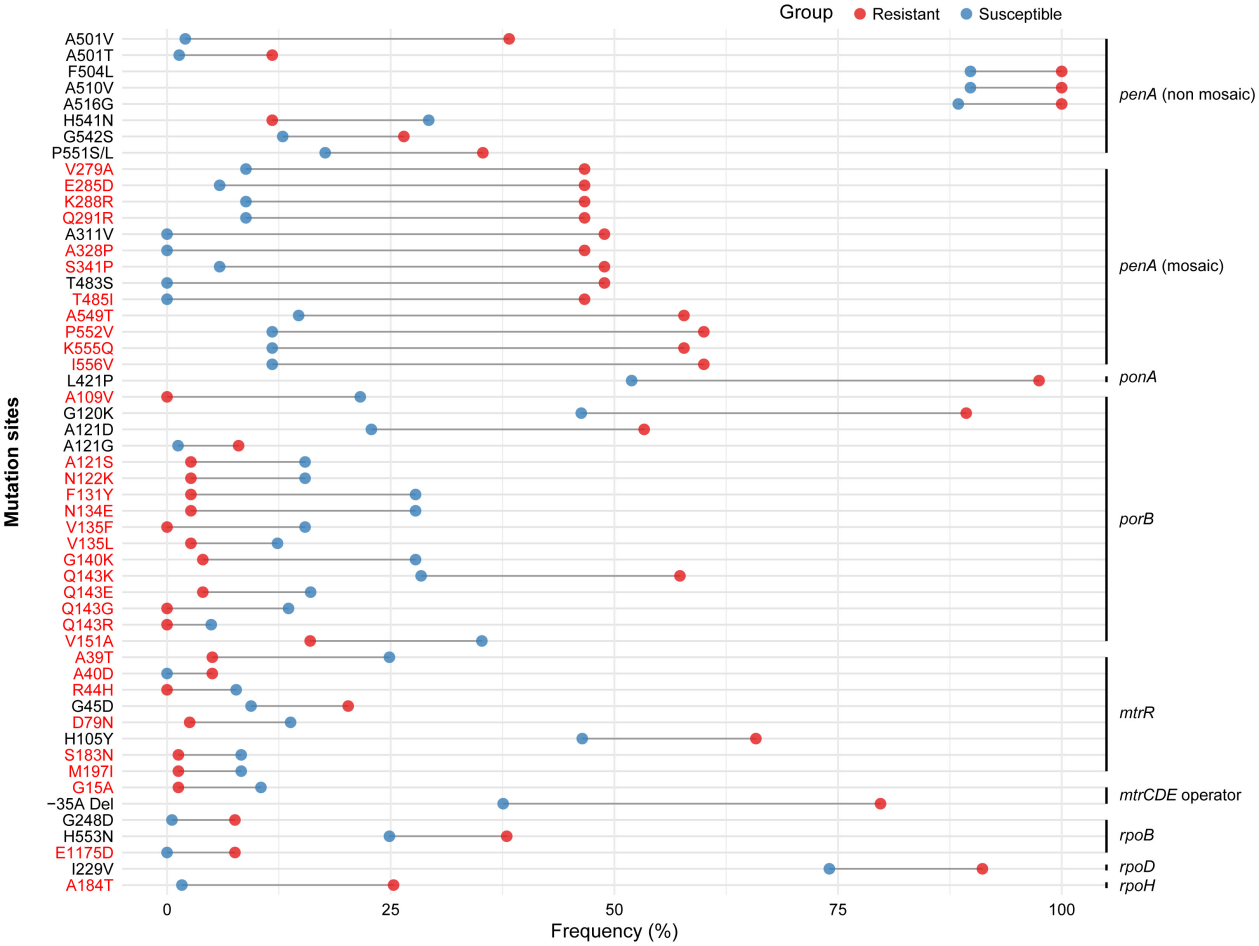
The frequency difference analysis of mutation sites. Resistant group in red, susceptible group in blue. Line length corresponds to the magnitude of intergroup difference.

The 260 strains were divided into ceftriaxone-resistant (CRO-R, *n* = 79) and ceftriaxone-susceptible (CRO-S, *n* = 181) groups. Amino acid frequencies in the “key regions” of each gene were statistically analyzed to assess the association between mutation sites and CRO resistance or susceptibility. Statistical analysis identified 53 distinct mutations with significant differences. These were considered potential genetic determinants of CRO resistance or susceptibility (Table 1). Cleveland dot plots were used to show the frequency differences of these mutations between resistant and susceptible groups, with newly identified mutations highlighted in red (Figure 2), including the following: mosaic PenA V279A, E285D, K288R, Q291R, A328P, S341P, T485I, A549T, P552V, K555Q, I556V, PorB A109V, A121S, N122K, F131Y, N134E, V135F, V135L, G140K, Q143K, Q143E, Q143G, Q143R, V151A, MtrR A39T, A40D, R44H, D79N, S183N, M197I, *mtrCDE* operator G15A, RpoB E1175D, RpoH A184T.

**Table 1:** Potential genetic determinants of ceftriaxone resistance or susceptibility.

**Mutation sites**	**CRO-R isolates**	**CRO-S isolates**	***P* value**	***q* value**
**PenA (non-mosaic)**				
A501V	13/34	3/147	1.55E-08	1.64E-07
A501T	4/34	2/147	1.20E-02	1.59E-02
F504L	34/34	132/147	3.83E-02	3.98E-02
A510V	34/34	132/147	3.83E-02	3.98E-02
A516G	34/34	130/147	2.42E-02	2.73E-02
H541N	4/34	43/147	2.52E-02	2.77E-02
G542S	9/34	19/147	4.92E-02	4.92E-02
P551S/L	12/34	26/147	2.42E-02	2.73E-02
**PenA (mosaic)**				
V279A	21/45	3/34	2.20E-04	4.33E-04
E285D	21/45	2/34	4.65E-05	1.12E-04
K288R	21/45	3/34	2.20E-04	4.33E-04
Q291R	21/45	3/34	2.20E-04	4.33E-04
A311V	22/45	0/34	2.10E-07	1.59E-06
A328P	21/45	0/34	5.07E-07	2.68E-06
S341P	22/45	2/34	2.16E-05	5.93E-05
T483S	22/45	0/34	2.10E-07	1.59E-06
T485I	21/45	0/34	5.07E-07	2.68E-06
A549T	26/45	5/34	8.73E-05	2.01E-04
P552V	27/45	4/34	9.88E-06	3.27E-05
K555Q	26/45	4/34	2.24E-05	5.93E-05
I556V	27/45	4/34	9.88E-06	3.27E-05
**PonA**				
L421P	77/79	94/181	2.69E-15	1.42E-13
**PorB1b**				
A109V	0/75	35/162	4.39E-07	2.68E-06
G120K	67/75	75/162	4.11E-11	1.09E-09
A121S	2/75	25/162	1.96E-03	3.24E-03
A121D	40/75	37/162	4.24E-06	1.61E-05
A121G	6/75	2/162	1.35E-02	1.71E-02
N122K	2/75	25/162	1.96E-03	3.24E-03
F131Y	2/75	45/162	6.47E-07	2.86E-06
N134E	2/75	45/162	6.47E-07	2.86E-06
V135F	0/75	25/162	3.90E-05	9.84E-05
V135L	2/75	20/162	1.09E-02	1.48E-02
G140K	3/75	45/162	4.13E-06	1.61E-05
Q143E	3/75	26/162	4.97E-03	7.32E-03
Q143G	0/75	22/162	1.42E-04	3.14E-04
Q143R	0/75	8/162	4.51E-02	4.59E-02
Q143K	43/75	46/162	2.02E-05	5.93E-05
V151A	12/75	57/162	1.57E-03	2.77E-03
**MtrR**				
A39T	4/79	45/181	1.72E-05	5.36E-05
A40D	4/79	0/181	9.90E-03	1.38E-02
R44H	0/79	14/181	3.79E-03	5.73E-03
G45D	16/79	17/181	2.56E-02	2.77E-02
D79N	2/79	25/181	2.58E-03	4.15E-03
H105Y	52/79	84/181	1.29E-02	1.66E-02
S183N	1/79	15/181	1.53E-02	1.84E-02
M197I	1/79	15/181	1.53E-02	1.84E-02
***mtrCDE*** **operator**				
G15A	1/79	19/181	5.47E-03	7.84E-03
−35A Del	63/79	68/181	1.83E-10	3.23E-09
**RpoB**				
G248D	6/79	1/181	3.62E-03	5.65E-03
H553N	30/79	45/181	2.39E-02	2.73E-02
E1175D	6/79	0/181	6.87E-04	1.30E-03
**RpoD**				
I229V	72/79	134/181	9.24E-04	1.69E-03
**RpoH**				
A184T	20/79	3/181	5.44E-09	7.20E-08

The mutation in mosaic *penA* is defined as a sequence composed of the amino acid residues with the highest frequency at each position relative to the mosaic *penA* of susceptible strain. All other mutations are described relative to FA1090. Statistical analysis: Mutation sites with significant differences were identified using two-tailed Fisher's exact tests with a significance threshold of *P* < 0.05. Multiple testing correction was applied via the Benjamini–Hochberg false discovery rate (FDR) method, with corrected *P* values (*q* values) reported at *q* < 0.05.

### Molecular mechanism analysis of ceftriaxone resistance in *Neisseria gonorrhoeae* through molecular typing

**Figure 3:**
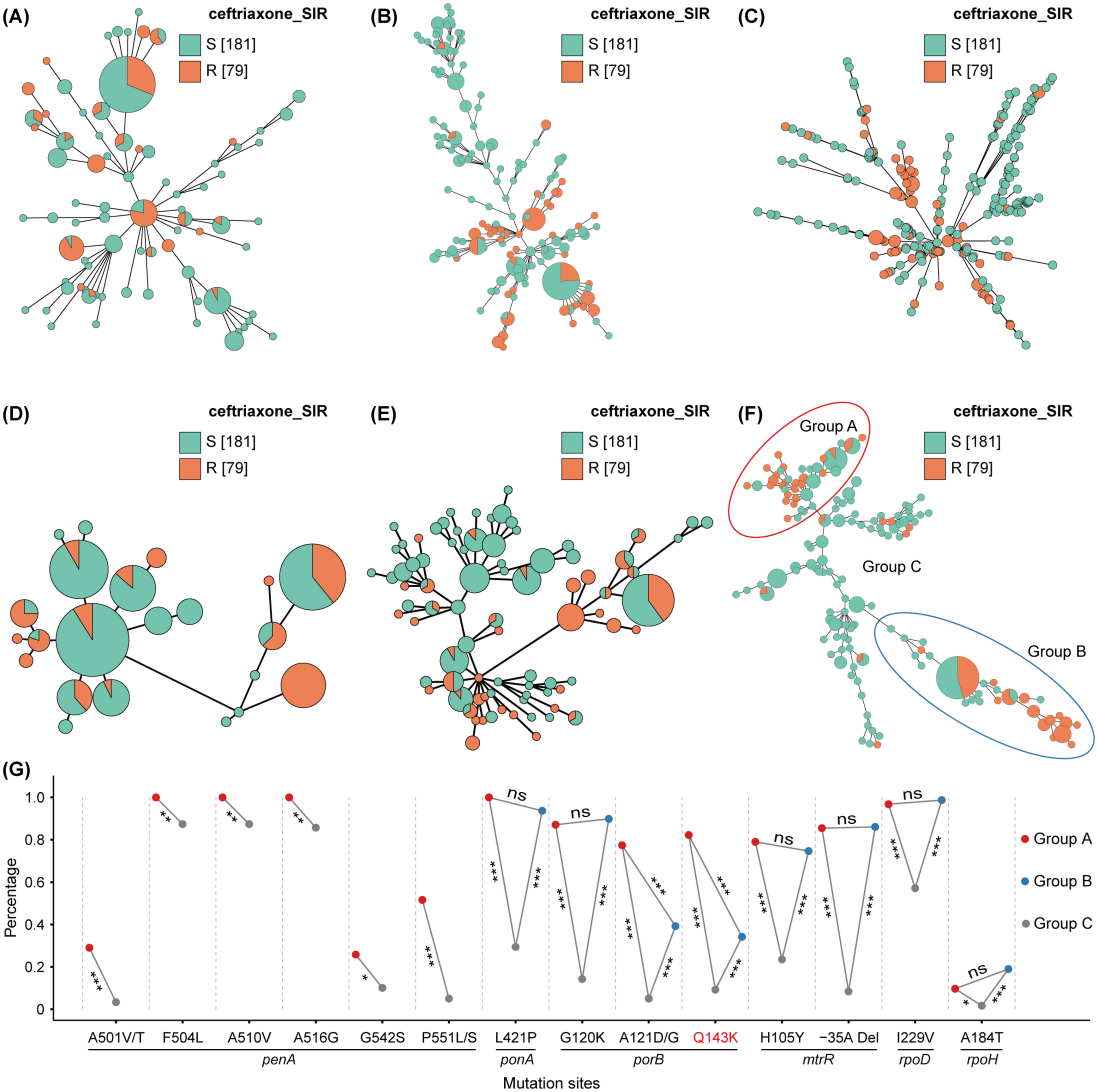
Molecular typing and mutation site difference analysis associated with ceftriaxone resistance. Orange indicates ceftriaxone resistance, and green indicates susceptibility. **(A)** MLST of the 7 housekeeping genes. **(B)** NG-STAR of the 7 resistance genes. **(C)** cgMLST of the 1,361 core genes. **(D)** Sequence typing of 20 sites in PenA. **(E)** Sequence typing of 14 common sites in PenA, PonA, PorB, and MtrR. **(F)** Sequence typing of 49 sites in PenA, PonA, PorB, MtrR, mtrCDE operator, *bla*TEM, RpoB, RpoD, and RpoH. **(G)** Comparison of the percentage of different mutation sites among the three groups in Figure **(F)**. For each mutation site, red, blue, and gray dots represent its percentage in Group A, B, and C, respectively. Lines between dots show comparisons between groups, with significance levels denoted as follows: ns (not significant, *q* ≥ 0.05), * (*q* < 0.05), ** (*q* < 0.01), and *** (*q* < 0.001).

To further investigate the molecular mechanisms of CRO resistance, we conducted a comparative analysis of various sequence typing methods. First, we compared MLST, NG-STAR, and cgMLST (core genome multilocus sequence) methods (Figures 3A–C). As expected, NG-STAR (based on 7 resistance genes) and cgMLST (using 1,361 core genes) outperformed MLST in differentiating resistant and susceptible strains. However, the numerous genes and countless sites obscure which ones are critical for CRO resistance.

Therefore, to identify which sites are crucial for CRO resistance, we compared the sequence typing of mutation sites across multiple genes. As shown in PenA sequence typing (Figure 3D), the sensitivity (percentage of resistant strains forming distinct branches) was only 36.7%, and the specificity (percentage of sensitive strains forming distinct branches) was only 12.7%. Therefore, PenA alone is insufficient for distinguishing CRO-R strains. In contrast, the combined sequence typing of PenA, PonA, PorB, and MtrR (Figure 3E) showed a sensitivity of 53.2% and a specificity of 57.5%, representing a clear improvement. Notably, sequence typing based on the 49 sites (Figure 3F, details in Supplementary Table 1) achieved a sensitivity of 68.4% and specificity of 77.3%, showing further improvement. Additionally, a large majority of non-mosaic resistant (Group A, red circle) and sensitive (Group C) strains are clustered by this method, with all mosaic resistant strains uniquely assigned to Group B (blue circle). Resistant strains in Groups A and B account for 89.9% of all resistant strains, demonstrating strong clustering efficacy.

The percentages of the previously identified resistance-associated mutations were compared among the three groups. The mutation sites showing significant differences, including PenA A501V/T, F504L, A510V, A516G, G542S, P551L/S; PonA L421P; PorB G120K, A121D/G, Q143K; MtrR H105Y; the−35A Del in the mtrR promoter; RpoD I229V; and RpoH A184T, are displayed in Figure 3G. These mutations effectively distinguished the clusters of resistant strains (Groups A and B) from sensitive strains (Group C), highlighting their critical role in differentiating CRO resistance and susceptibility. Unlike other mutations, PorB A121D/G and the first-identified PorB Q143K (red font) also show significant differences between Groups A and B, suggesting distinct mutation profiles in non-mosaic and mosaic resistant strains. Overall, these findings provide further insight into the molecular mechanisms of ceftriaxone resistance in *N. gonorrhoeae*.

### Analysis of the correlation between mutation frequencies at key PenA sites and MLST types

Based on the above analysis, 20 distinct mutation sites in PenA were identified. Among them, H541N was associated with susceptibility, while F504L, A510V, and A516G were commonly mutated in both resistant and susceptible strains, and thus excluded. The final key PenA mutation sites were identified as A501V/T, G542S, and P551L/S in non-mosaic alleles, along with 13 sites in mosaic alleles. The mutation frequency at key PenA sites and the occurrence of mosaic penA alleles were statistically analyzed for each MLST type. The mutation frequency coefficient (k = 1) was defined as the overall mutation rate at each site, calculated by dividing the total number of mutations at each site by the total number of strains (counting mosaic and non-mosaic strains separately). The threshold of k = 2 was set for identifying high-frequency STs.

Among 8,325 strains (non-mosaic = 7,678; mosaic = 647), 129 STs exceeding the threshold were identified as high-frequency STs. A total of 34 MLST types with ≥ 5 isolates were defined as statistically significant high-frequency STs. 33 STs were identified from non-mosaic alleles, including ST7363, ST7822, ST1579, ST1583, ST1901, ST10314, ST7827, ST1893, ST11981, ST11706, ST7371, ST1600, ST1903, ST7823, ST13489, ST1902, ST7365, ST7367, ST8123, ST10313, ST11230, ST7360, ST14422, ST1927, ST13143, ST12982, ST1920, ST8153, ST16414, ST1590, ST6714, ST11231, and ST9902. The high-frequency STs corresponding to mosaic sites must also belong to the high-frequency mosaic types, resulting in the identification of only two types: ST7363 and ST13734.

**Figure 4:**
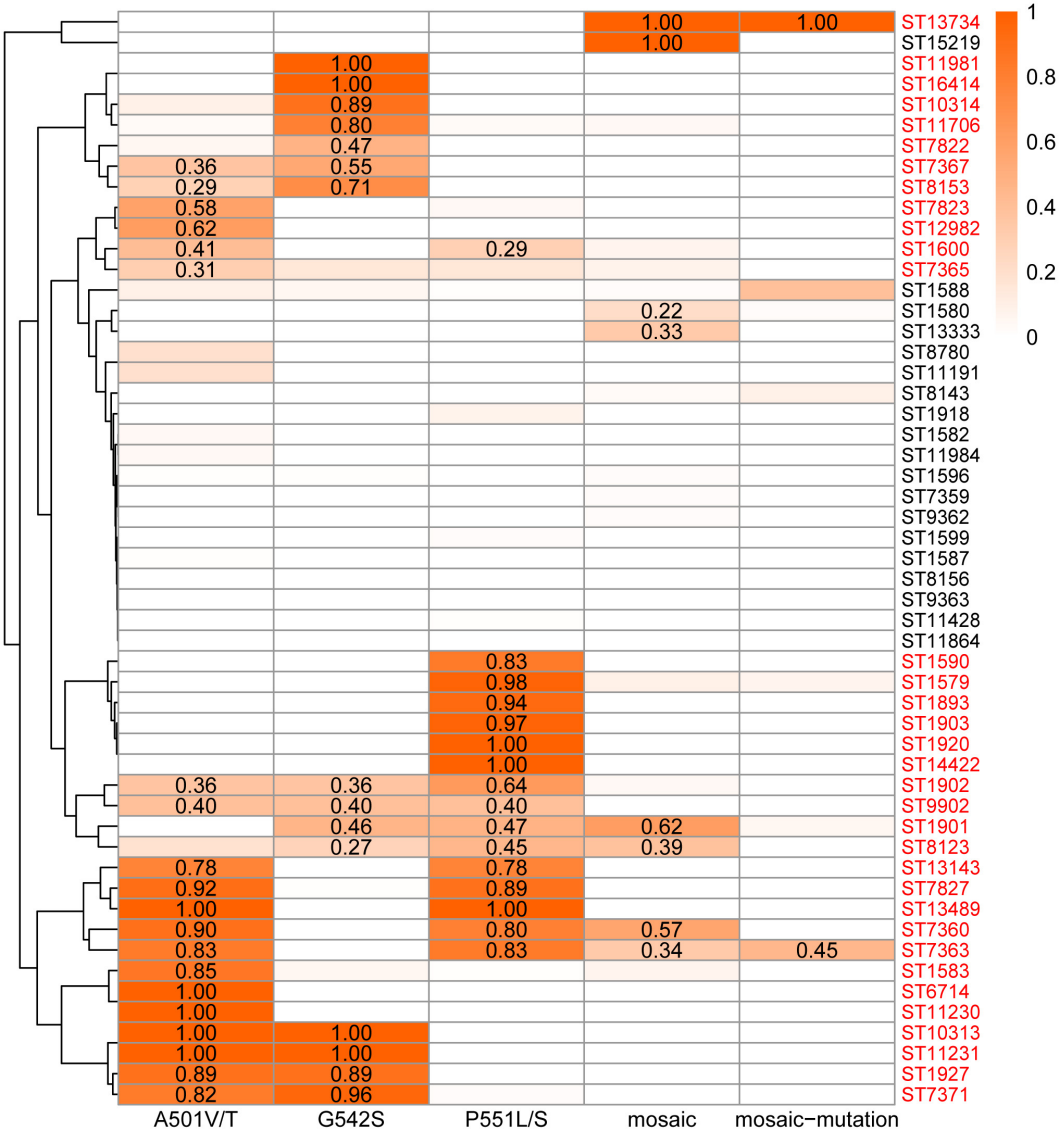
Clustering heatmap of PenA Mutations and MLST types. A gradient color scale from white (0) to orange (1) indicates the mutation frequency values across the range of 0 to 1. Use red font for high-frequency MLST types, and display the exact frequency values that exceed the thresholds.

Clustering analysis of mutation frequencies at key PenA sites was performed for all MLST types with ≥ 5 isolates (Figure 4). For ST1902 and ST9902, all three non-mosaic loci are high-frequency mutations. For ST1901, ST8123, and ST7360, two non-mosaic loci are high-frequency mutations, and their mosaic frequencies exceed the threshold. ST7363 is the only MLST type that belongs to both high-frequency non-mosaic and mosaic groups. Detailed mutation sites, frequencies, and the identifiers of the 129 STs are provided in Supplementary Table 3.

**Figure 5:**
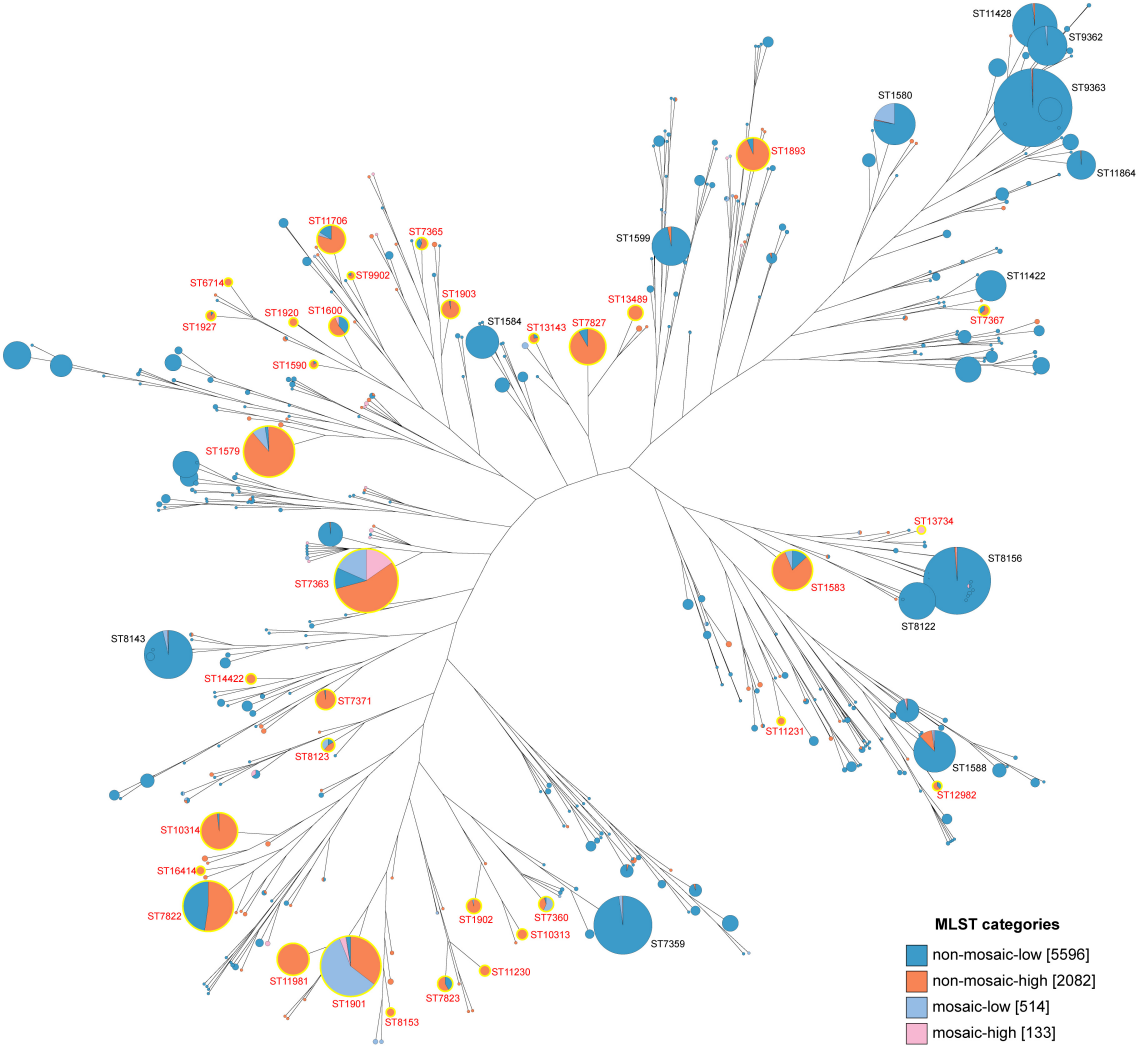
Neighbor-joining tree of MLST for 8,325 isolates. Each circle represents an MLST type, with size indicating the number of isolates. High-frequency types are displayed in orange (non-mosaic) and pink (mosaic), while low-frequency types are shown in dark blue (non-mosaic) and light blue (mosaic).

A neighbor-joining tree based on MLST was constructed to show the evolutionary relationships among STs (Figure 5). This analysis systematically illustrated the distribution patterns of mutation frequencies at key PenA sites across different MLST types. Strains with key mutation sites were classified as “non-mosaic-high” or “mosaic-high,” while the others were categorized as “non-mosaic-low” or “mosaic-low.” From this, we can observe that the mutation frequencies at key PenA sites are highly associated with MLST types. 34 high-frequency STs are labeled in red and highlighted in yellow, while some types show very low or even zero mutation frequency despite having large strain numbers (*n* > 100, labeled in black).

**Figure 6:**
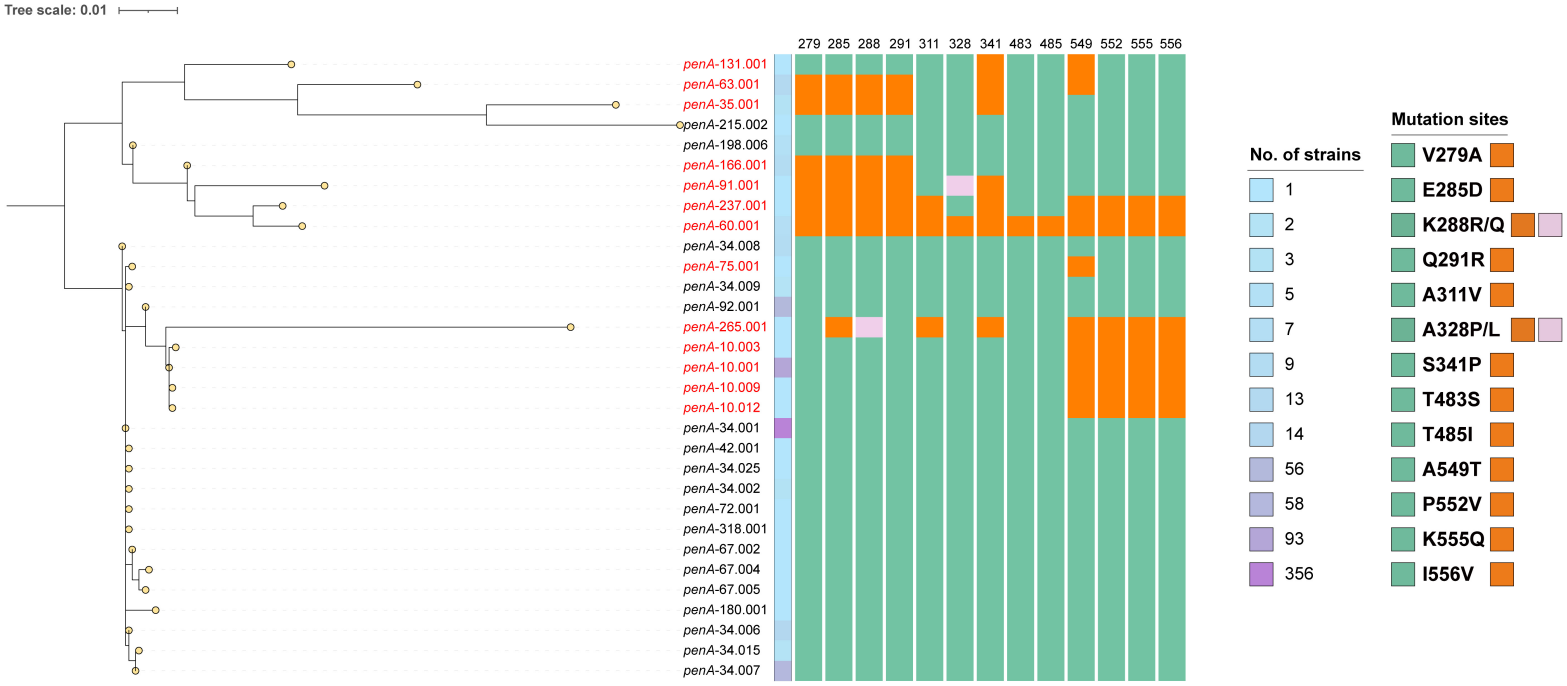
The phylogenetic analysis of 31 mosaic *penA* alleles from 647 isolates. Midpoint-rooted for visualization only. Mosaic *penA* alleles linked with resistance-associated mutations are shown in red font. The number of isolates is indicated by a gradient from light blue to purple. Pre-mutation amino acids are colored green, while post-mutation amino acids are colored orange or pink.

Not all mosaic strains are CRO-R (Mlynarczyk-Bonikowska et al., 2022), but specific mosaic *penA* alleles, particularly in FC428-like strains, are strongly associated with CRO-R (Xiu et al., 2025). Therefore, for 647 *penA* mosaic strains, we must not only analyze the relationship between mutation sites and MLST types but also investigate the link between mutation sites and *penA* alleles. The phylogenetic tree based on 31 mosaic *penA* alleles from 647 isolates divided them into two groups (Figure 6). One group contained 1 to multiple resistance-associated mutations, with *penA*-60.001 harboring all 13 mutations and being significantly associated with CRO-R (Adamson et al., 2024). The other group lacked resistance-associated mutations, such as the most prevalent allele *penA*-34.001. This suggests that the 13 previously identified key mosaic mutation sites are highly correlated with mosaic *penA* alleles and CRO-R.

To evaluate the utility of the 34 high-frequency STs in ceftriaxone resistance surveillance, we validated them using 611 CRO-DS strains (MIC > 0.064 mg/L; see Supplementary Table 2 for background information) (Lindberg et al., 2007; Whiley et al., 2010; Lin et al., 2021) from all 23,381 isolates. As shown in Table 2, 88.38% (540/611; 95% CI: 85.85–90.91%) of CRO-DS strains belonged to 34 high-frequency STs, compared to 33.09% (2,755/8,325; 95% CI: 32.09–34.09%) of background strains. A chi-square test was used to compare the distribution of the 34 high-frequency STs between the two groups. The results showed that CRO-DS strains were significantly enriched in the 34 high-frequency STs (χ^2^ = 747.48, df = 1, *P* < 0.0001). Relative risk analysis demonstrated that CRO-DS strains were 2.67 times more likely to occur in high-frequency STs than background strains (RR = 2.67, 95% CI: 2.56–2.79). In summary, the 34 high-frequency STs demonstrated an extremely significant positive correlation with CRO-DS (*P* < 0.0001). 88.38% of CRO-DS strains were concentrated in the 34 high-frequency STs, which constitute only 7.23% (34/470) of the MLST types in the background strains. This indicates that the 34 high-frequency STs are strong predictors of CRO-DS risk. Moreover, strains belonging to the 34 high-frequency STs account for 33.09% of background strains, suggesting that approximately one-third of *N. gonorrhoeae* may carry a notably higher risk of CRO-DS. Meanwhile, the major NG-STAR and NG-MAST types corresponding to the 34 high-frequency STs are shown in Supplementary Figure 3, including NG-STAR ST90 (Demczuk et al., 2017) and NG-MAST ST1407 (Gianecini et al., 2019), both reported in association with CRO-DS. Therefore, continuous monitoring of 34 high-frequency STs and their corresponding NG-STAR and NG-MAST types is of great significance.

**Table 2:** Distribution of 34 high-frequency MLST types among CRO-DS strains and background strains.

**Group**	**In 34 high-frequency STs**	**Not in 34 high-frequency STs**	**Total**	**Proportion (95% CI)**
CRO-DS strains	540	71	611	88.38% (85.85%-90.91%)
Background strains	2,755	5,570	8,325	33.09% (32.09%-34.09%)

Statistical analysis: χ^2^ = 747.48, df = 1, *P* < 0.0001; RR = 2.67 (95% CI: 2.56–2.79).

## Discussion

Given the increasing global reports of CRO-DS isolates (Lin et al., 2022; Fifer et al., 2024; Xiu et al., 2024), the importance of researching first-line and last-line ceftriaxone resistance mechanisms and enhancing surveillance cannot be overstated. This study utilized genomic and resistance data from 23,381 isolates (as of November 18, 2024) in pubMLST, the largest global *N. gonorrhoeae* database. CRO-R and CRO-S strains were used to investigate the molecular mechanisms of CRO resistance. Strains lacking cephalosporin resistance data (forming a distinct dataset from the CRO-DS strains used for validation) were used to analyze relationships between mutation frequencies at key PenA sites, MLST types, and CRO-DS.

Pan-genome analysis identified two gene clusters, A and B, associated with GGI. The GGI secretes DNA into the environment via a type IV secretion system (T4SS) independent of direct cell-cell contact (Hamilton et al., 2005), while *N. gonorrhoeae* can acquire environmental DNA through natural transformation. This dual mechanism drives the rapid spread of antibiotic resistance. Notably, the GGI has been reported to significantly correlate with CRO-R (Harrison et al., 2016). These findings further suggest potential links between the A/B clusters and CRO-R, underscoring the importance of pan-genome analysis in studying gonococcal antibiotic resistance.

This study systematically identified 53 mutation sites with significant differences in genes associated with ceftriaxone resistance (CRO-R) and susceptibility (CRO-S) in *N. gonorrhoeae* through literature review, statistical analysis, and protein structure/interaction assessments. Among these, 33 mutations were newly reported, with the Q143K mutation in PorB (expanded from 10aa to 49aa by structural analysis) being highlighted for the first time as significantly linked to ceftriaxone resistance. Based on these mutation sites, we constructed a minimum spanning tree (Figure 3F) to investigate the molecular mechanisms of CRO resistance, which effectively segregated CRO-R and CRO-S strains. However, this typing method still has limitations. First, the limited number of mosaic strains (*n* = 79) and mosaic alleles (*n* = 12) may lead to incomplete identification of key mosaic mutation sites. Second, the largest branch of mosaic strains includes 19 susceptible strains (*penA*-34.001) and 16 resistant isolates (*penA*-34.001, *n* = 9; *penA*-42.001, *n* = 4; *penA*-34.009, *n* = 2; *penA*-34.01, *n* = 1), which could not be distinguished by these mutation site combinations, limiting further improvements in sensitivity and specificity. This suggests that there may be other, yet unidentified resistance mechanisms. Since the mechanisms of ceftriaxone resistance in *N. gonorrhoeae* involve a complex, multi-gene determined process, further rationally designed experiments are therefore needed for phenotypic validation. Nevertheless, these findings provide new insights for identifying CRO-R strains and understanding the molecular mechanisms of CRO resistance.

ST7363 and ST1901 are the most widely reported STs associated with CRO-DS globally (Thomas et al., 2021), likely due to their high global prevalence and the high-frequency mutations/accumulation at key PenA sites coupled with a remarkably high mosaic frequency. ST7363 exhibits 83% mutation frequency at non-mosaic sites A501V/T and P551L/S, with mosaic frequency and mosaic site mutation frequency at 34% and 45%, respectively. ST1901 shows 46% and 47% mutation frequencies at non-mosaic sites G542S and P551L/S, alongside a mosaic frequency of 62%. Although CRO resistance in *N. gonorrhoeae* is a complex multigenic process, analysis of mutation frequencies at key PenA sites still partially explains the molecular mechanisms underlying CRO resistance in ST7363 and ST1901.

Our study revealed for the first time that mutation frequencies at key PenA sites are highly correlated with MLST types, with substantial variation observed across different MLST types. Validation using 611 CRO-DS strains confirms that MLST types with high-frequency mutations at these key sites are more prone to develop CRO-DS strains. We know that different MLST types represent distinct genetic lineages. Therefore, we hypothesize that high-frequency and low-frequency mutation groups at key PenA sites may differ in their DNA replication and repair mechanisms, capacity to take up or transform exogenous DNA, or the local sequence context of the *penA* and other resistance-related genes. Studies have shown that local sequence context strongly impacts DNA replication accuracy *in vivo* (Schroeder et al., 2016). This may represent an important avenue for future research into the mechanisms underlying why specific MLST types are more prone to developing CRO-DS strains. However, considerable experimentation is still required to validate these hypotheses.

Our findings are strongly supported by previous reports. ST7363, ST1901, ST1903, ST7365, and ST7360 are widely reported to be associated with CRO-DS (Li et al., 2021; Liao et al., 2023; Tang et al., 2023; Laumen et al., 2025). Notably, in Shenzhen, China, ST11231, ST7365, and ST8123 exhibited notable CRO-R rates of 35.71%, 28.30%, and 13.04% during 2019–2020, while these STs exhibited 100% susceptibility to CRO in 2014–2018. The authors defined ST11231, with the highest CRO-R rate, as a key target for future surveillance (Wang et al., 2023). Separately, 8 of 10 CRO-R strains detected in the UK during 2021–2022 belonged to ST8123 (Day et al., 2022). All reported STs fall within the 34 high-frequency STs identified in our study. Additionally, among the 95 types exceeding the threshold but excluded from the 34 high-frequency STs, some, like ST16406 (Ouk et al., 2023; Fifer et al., 2024) and ST13871 (Trinh et al., 2022), are still widely reported to be associated with CRO-R. Therefore, it is necessary to increase the number of strains to verify whether these types belong to high-frequency STs. We recommend prioritizing these 34 STs for monitoring CRO resistance in *N. gonorrhoeae* and encourage researchers to adopt similar approaches using diverse isolates, along with more mutation sites, to identify additional high-frequency STs.

## Data Availability

The genomic data used in this study were all obtained from the PubMLST database (https://pubmlst.org/bigsdb?db=pubmlst_neisseria_isolates&page=query&genomes=1). Genomic background information and additional data supporting the findings of this study are available in the text and Supplementary material.

## Supplementary material

Supplementary Figure 1 The phylogenetic tree of 260 isolates based on core SNPs.

Supplementary Figure 2 Structural analysis of PenA, PorB, and MtrR.

Supplementary Figure 3 The correspondence between MLST, NG-STAR, and NG-MAST.

Supplementary Table 1 Background information and mutation sites of 260 strains.

Supplementary Table 2 Background information of 8,325 background strains and 611 CRO-DS strains.

Supplementary Table 3 Mutation sites and frequencies in PenA from 8,325 strains and identifiers of 129 MLST types.
