# Supplementary material for: Genetic identification of mutations and MLST types associated with decreased susceptibility to ceftriaxone in Neisseria gonorrhoeae
Source: Front Microbiol. 2026 Jan 21;16:1728860. doi: 10.3389/fmicb.2025.1728860 (PMC12868221; doi:10.3389/fmicb.2025.1728860)
Supplement: Supplementary Figure 1 — The phylogenetic tree of 260 isolates based on core SNPs. [file Data_Sheet_1.pdf]

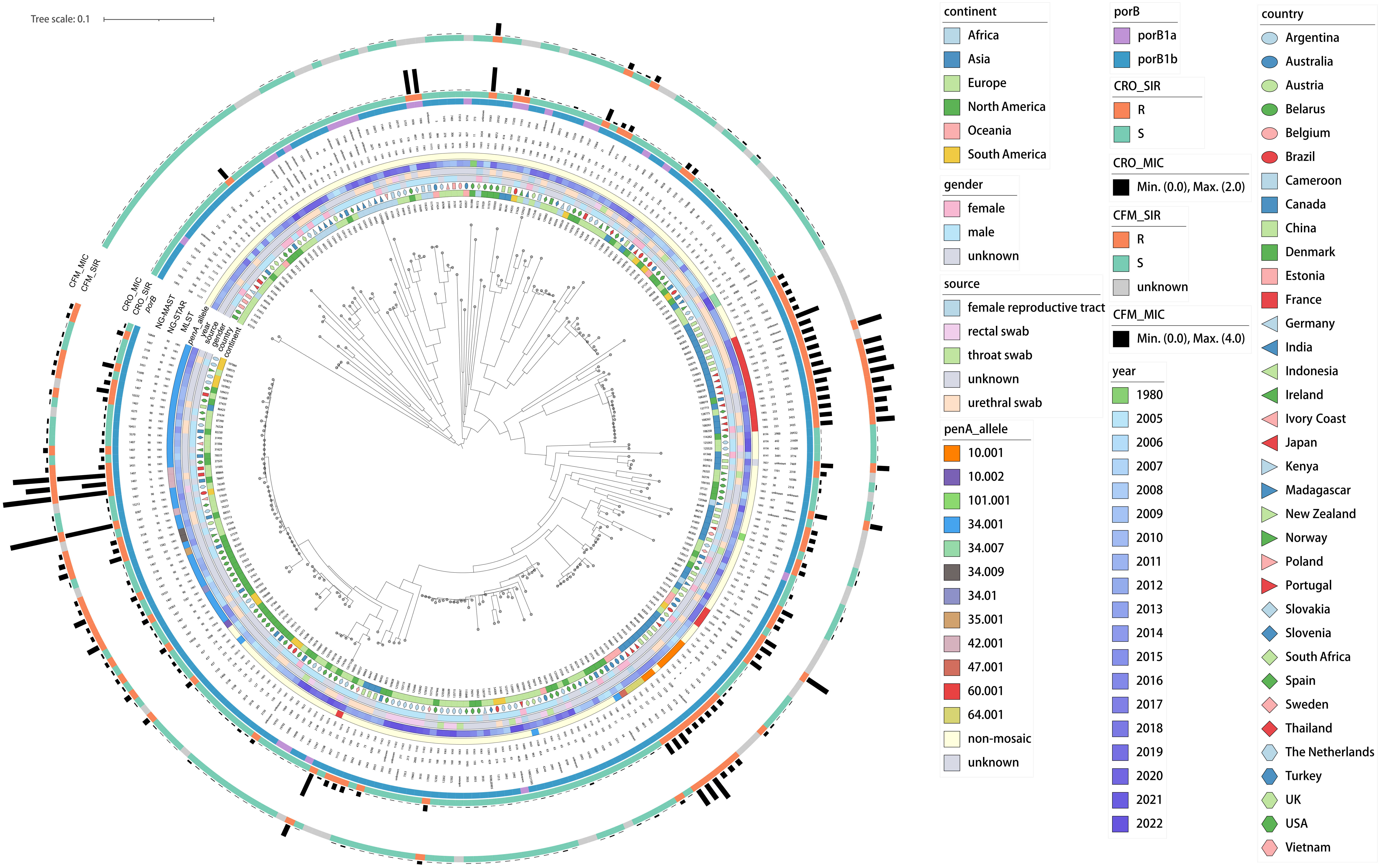

Supplementary Figure 1. The phylogenetic tree of 260 isolates based on core SNPs. Midpoint-rooted for visualization only. Countries are represented by different colors and shapes, years are shown with gradient colors, MIC values are displayed as bar plots. MLST, NG-STAR, and NG-MAST are labeled as text, and additional features are indicated by colored squares.
