# Supplementary material for: Genetic identification of mutations and MLST types associated with decreased susceptibility to ceftriaxone in Neisseria gonorrhoeae
Source: Front Microbiol. 2026 Jan 21;16:1728860. doi: 10.3389/fmicb.2025.1728860 (PMC12868221; doi:10.3389/fmicb.2025.1728860)
Supplement: Supplementary Figure 2 — Structural analysis of PenA, PorB, and MtrR. [file Data_Sheet_2.pdf]

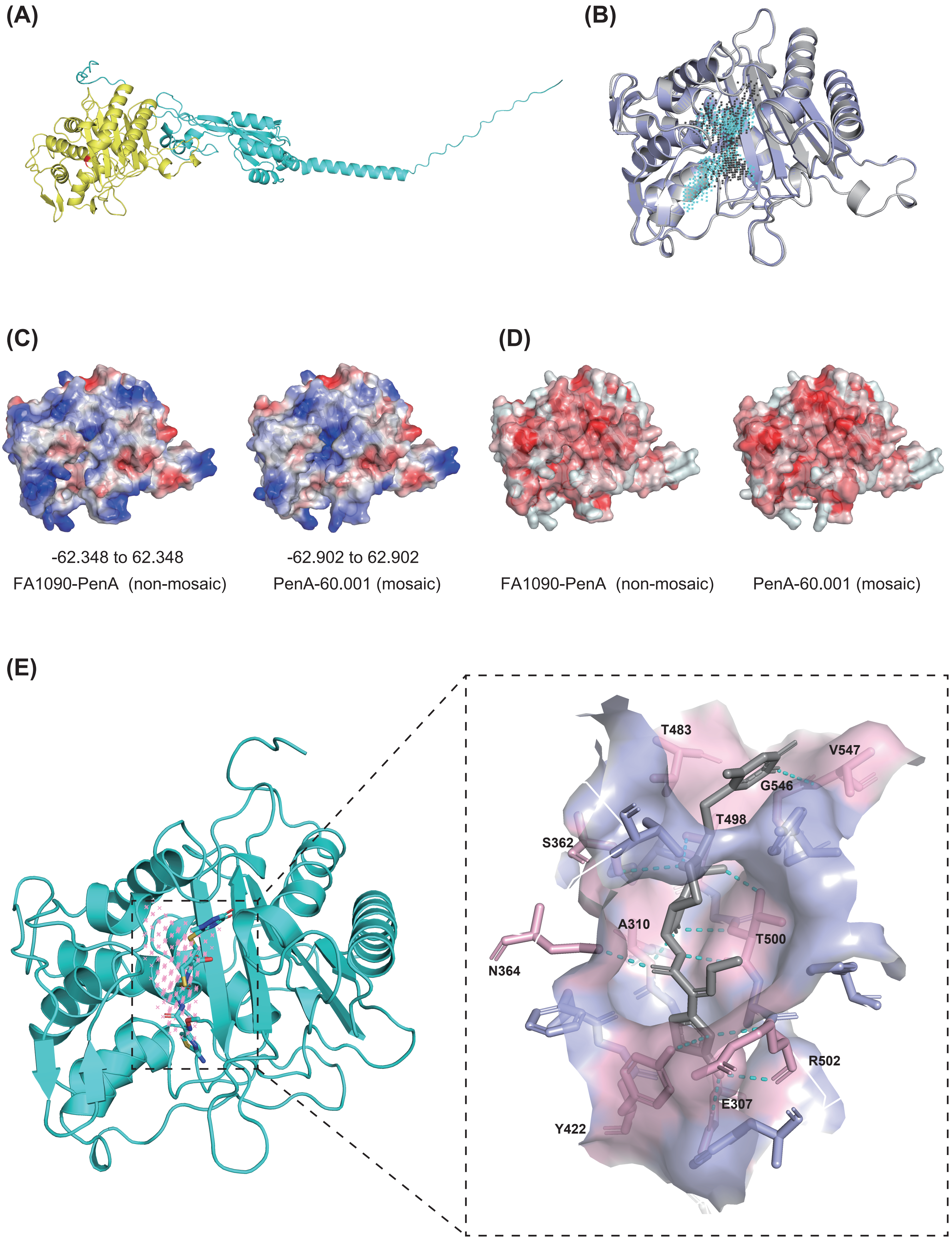
(A)
(B)
(C)
(D)
-62.348 to 62.348
FA1090-PenA (non-mosaic)
-62.902 to 62.902
PenA-60.001 (mosaic)
FA1090-PenA (non-mosaic)
PenA-60.001 (mosaic)
(E)
T483
V547
G546
T498
S362
A310
T500
N364
R502
E307
Y422

(F)

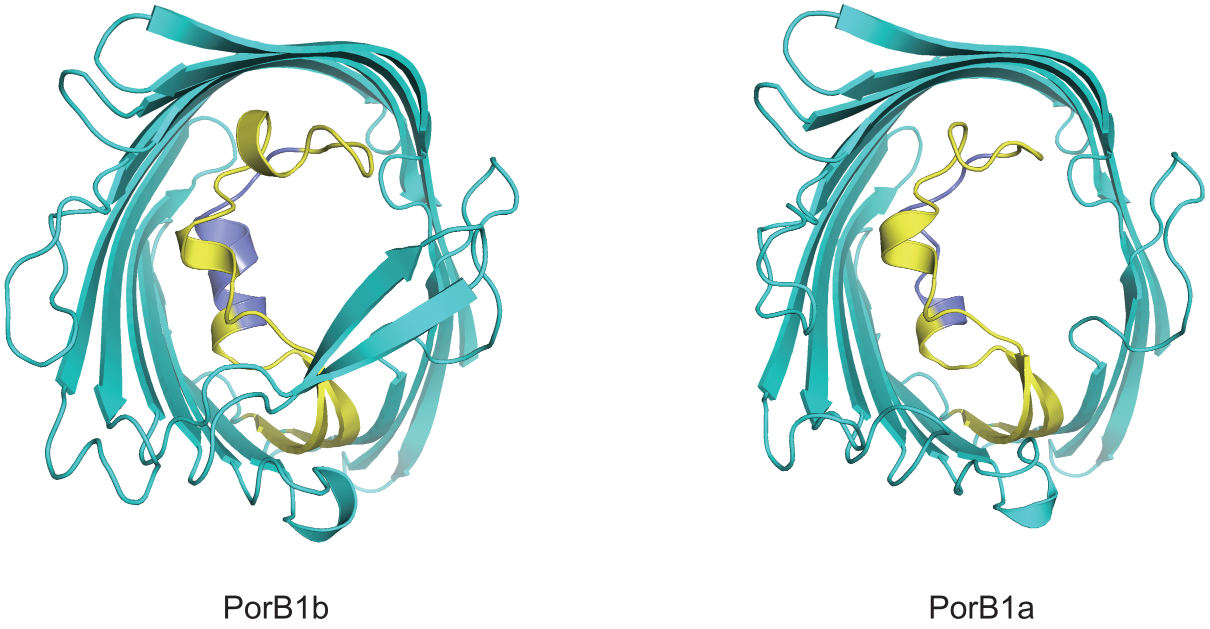
PorB1b
PorB1a

(G)

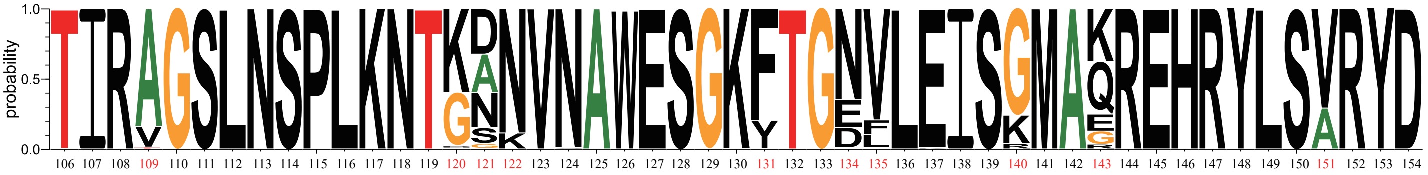
probability
1.0
0.5
0.0
106 107 108 109 110 111 112 113 114 115 116 117 118 119 120 121 122 123 124 125 126 127 128 129 130 131 132 133 134 135 136 137 138 139 140 141 142 143 144 145 146 147 148 149 150 151 152 153 154

(H)

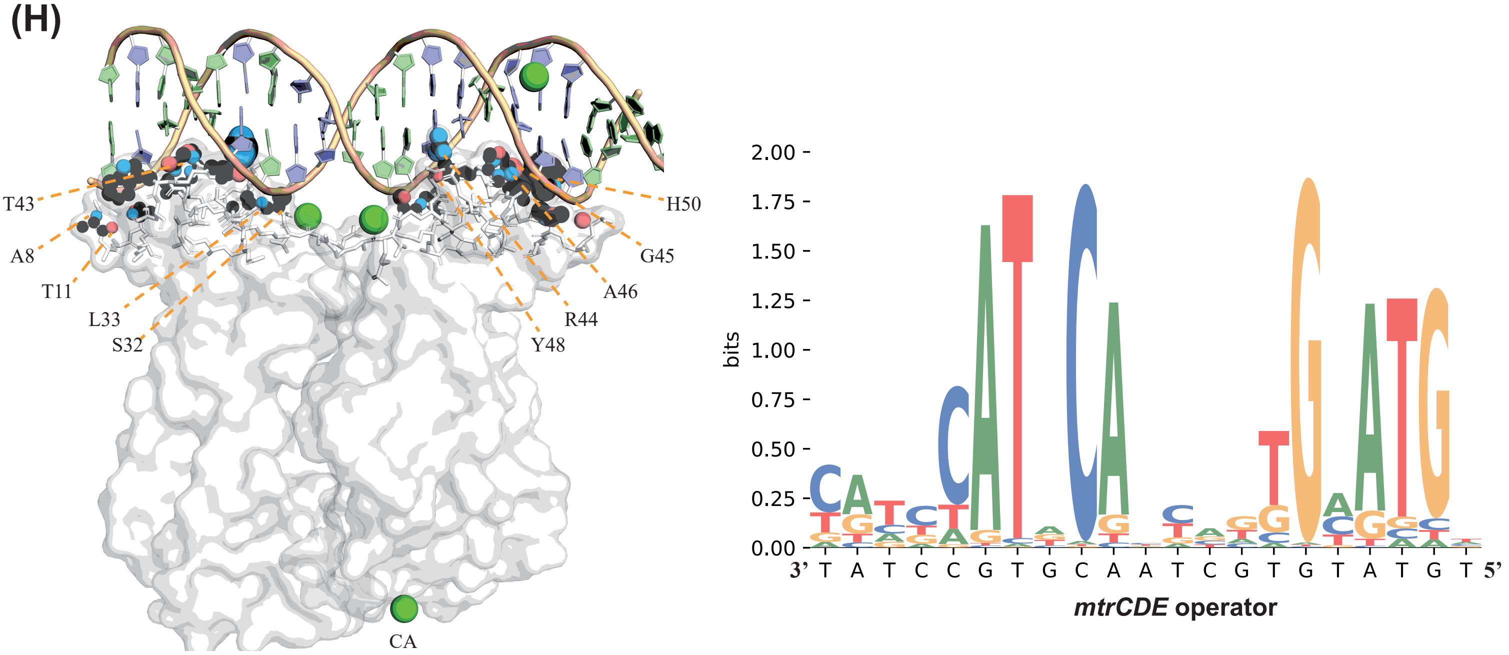
T43
A8
T11
L33
S32
Y48
R44
A46
G45
H50
CA
bits
2.00
1.75
1.50
1.25
1.00
0.75
0.50
0.25
0.00
3' T A T C C G T G C A A T C G T G T A T G T 5'
mtrCDE operator

Supplementary Figure 2. Structural analysis of PenA, PorB, and MtrR. **(A)** The 3D structure of FA1090-PenA, with Q241-L564 shown in yellow, the catalytic active site S310 highlighted in red, and other regions displayed in blue. **(B)** Structural alignment of non-mosaic PenA (FA1090-PenA, gray; active pocket, black dotted) and mosaic PenA (PenA 60.001, light blue; active pocket, cyan dotted). **(C)** Surface charge analysis of non-mosaic and mosaic PenA. Negative charge, red; neutral, white; positive charge, blue. **(D)** Surface hydrophobicity analysis of non-mosaic and mosaic PenA. Redder regions indicate higher hydrophobicity, while whiter regions indicate lower hydrophobicity. **(E)** Interaction of PenA with CRO. The active pocket is shown in pink dots, hydrogen bonds as blue dashed lines, and CRO-interacting residues (labeled) in pink. **(F)** Structure of porin PorB1b and PorB1a. The purple region represents the 10 amino acids in the PorB defined by NG-STAR, while the yellow region indicates the 49 amino acids located in the core variable region of PorB defined in this study. **(G)** Conservation analysis of the 49 amino acids core variable region in PorB1b. The red-marked sites are those identified in this study as associated with CRO resistance or susceptibility. **(H)** Protein-DNA binding specificity analysis of MtrR and mtrCDE operator. Orange dashes mark 10 interacting residues; green sphere indicates calcium ion. Right panel x-axis shows the 21mer operator sequences; larger nucleotides in plot indicate stronger MtrR binding specificity.
