# Supplementary material for: Genetic identification of mutations and MLST types associated with decreased susceptibility to ceftriaxone in Neisseria gonorrhoeae
Source: Front Microbiol. 2026 Jan 21;16:1728860. doi: 10.3389/fmicb.2025.1728860 (PMC12868221; doi:10.3389/fmicb.2025.1728860)
Supplement: Supplementary Figure 3 — The correspondence between MLST, NG-STAR, and NG-MAST. [file Data_Sheet_3.pdf]

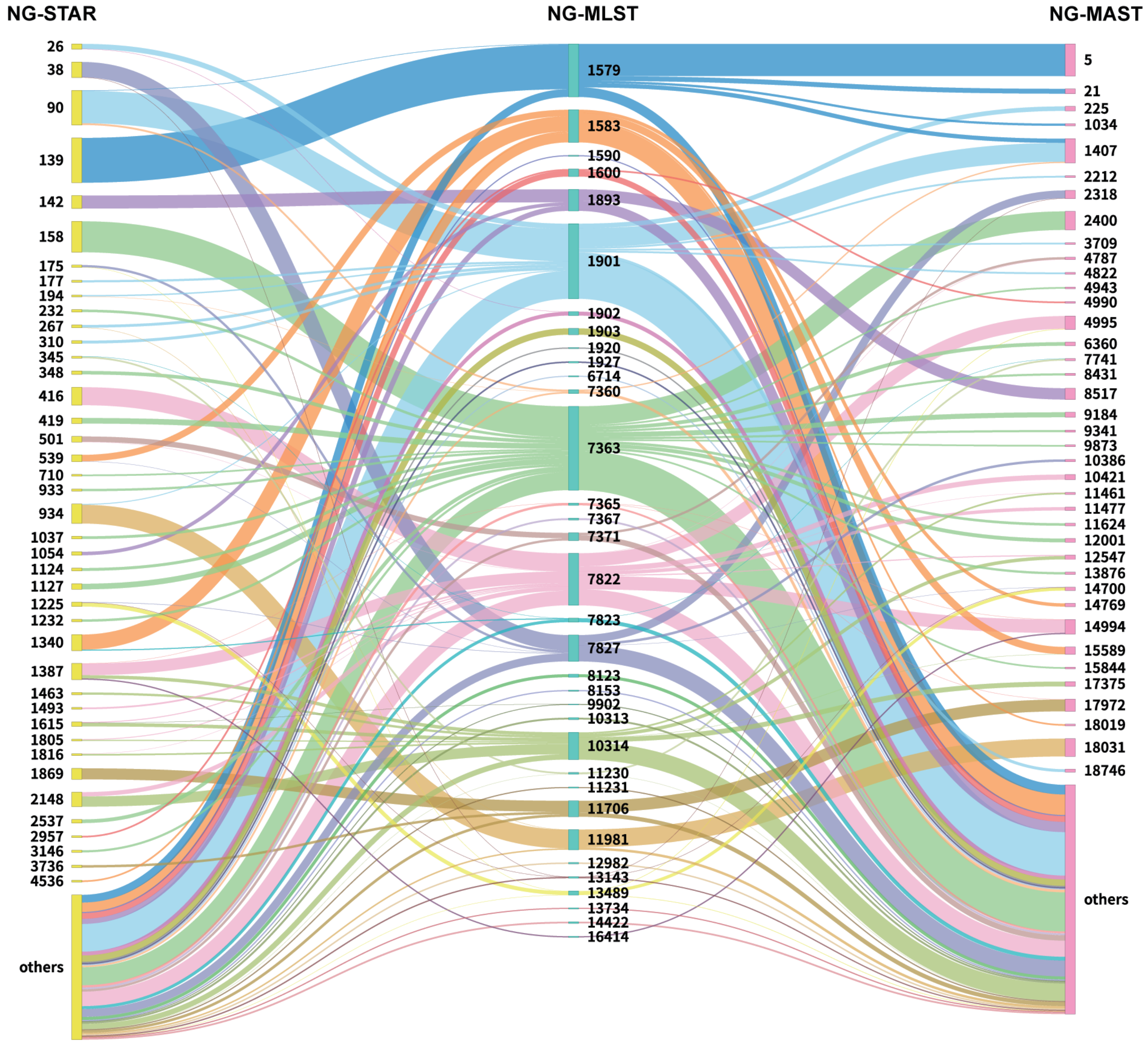

Supplementary Figure 3. The correspondence between MLST, NG-STAR, and NG-MAST. For NG-STAR and NG-MAST types, only those with counts ≥ 10 are shown; types with counts <10 are grouped under "others". All type identifiers across the three typing methods are listed in ascending order from top to bottom.
